# Differential Responses of Dimorphic Seeds and Seedlings to Abiotic Stresses in the Halophyte *Suaeda salsa*

**DOI:** 10.3389/fpls.2021.630338

**Published:** 2021-04-12

**Authors:** Hao Zhang, Mingfang Hu, Hongyuan Ma, Li Jiang, Zhenyong Zhao, Jinbiao Ma, Lei Wang

**Affiliations:** ^1^State Key Laboratory of Desert and Oasis Ecology, Xinjiang Institute of Ecology and Geography, Chinese Academy of Sciences, Urumqi, China; ^2^University of Chinese Academy of Sciences, Beijing, China; ^3^Northeast Institute of Geography and Agroecology, Chinese Academy of Sciences, Changchun, China

**Keywords:** germination index, halophyte, salt stress, seed heteromorphism, seedling growth, *Suaeda salsa*

## Abstract

The period between seed germination and seedling establishment is one of the most vulnerable stages in the life cycle of annuals in the saline environments. Although germination characteristics of *Suaeda salsa* seeds have been reported, the comparative germination patterns of dimorphic seeds and seedling growth to different abiotic stresses remain poorly understood. In this study, germination responses of dimorphic seeds to light and temperature were compared. Meanwhile, responses of dimorphic seeds and thereafter seedlings of *S. salsa* to different concentrations of NaCl and Na_2_SO_4_ were also tested. The results showed that the light did not significantly affect germination percentage of brown seeds, but significantly promoted germination of black seeds. Brown seeds could reach high germination percentage over a wide temperature range, however, germination of black seeds gradually increased with the increase of temperature. Brown seeds had higher germination percentage and velocity than black seeds under the same salt conditions. However, black seeds had higher recovery germination than brown seeds when transferred to deionized water. Young seedlings had lower salt tolerance than germinating seeds. At the same concentrations, Na_2_SO_4_ had stronger inhibitory effect on seed germination and seedling growth than NaCl. This study comprehensively compared germination traits of dimorphic seeds and seedling growth of *S. salsa*, and then developed a conceptual model to explain their adaptation to harsh saline environment.

## Introduction

*Suaeda salsa*, an annual halophyte, grows in saline and alkaline soils on lake shores and beaches of Asia and Europe (Zhu et al., 2003). This plant can accumulate high amount of salt in aboveground parts (Zhao, 1991; Wang et al., 2020). *S. salsa* has been studied for its potential in phytoremediation of saline soils and as gourmet vegetable, edible oil, and animal feed (Song and Wang, 2015; Shang et al., 2020). The fresh leaves of *S. salsa*, traditionally used as vegetable, have high nutritional value. The contents of protein, crude fiber, and vitamin C in fresh branches are 2.3, 63.7, and 13 mg kg^–1^, respectively. The dry seeds of *S. salsa* contain ca. 20% edible oil and the unsaturated fatty acids account for >90%. Furthermore, *S. salsa* is a promising model plant to study salt tolerance (Song and Wang, 2015).

*Suaeda salsa* produces two types of seeds on a single plant (Zhao et al., 2004). Dimorphic seeds are not only differ in color and morphology, but also differ in dormancy and germination (Li et al., 2005, 2008). Brown seeds are non-dormant and have higher germination percentage than black seeds in salinity, which regulated by the ABA and GA homeostases (Li et al., 2016). Though several studies have been conducted on the salt tolerance of *S. salsa* seeds during germination (Li et al., 2005, 2016), there are few studies concerning salt tolerance of young seedlings grown from dimorphic seeds and NaCl is generally the only one kind of salt used in the experiment (Duan et al., 2007; Song et al., 2008). The shoot length is more sensitive to high salinity for *S. salsa* seedlings grown from black seeds than that from brown seeds (Song et al., 2008). Duan et al. (2007) tested the effect of different types of salinity on germination inhibition of *S. salsa* and found that the effect order is MgCl_2_ > Na_2_SO_4_ > Na_2_CO_3_ > NaCl > MgSO_4_. Seedling growth is accelerated by the low concentration of salinity (0.05–0.1 mM). However, the seed type of *S. salsa* used in this experiment is not mentioned. Meanwhile, there is no comprehensive analysis on germination responses of dimorphic seeds of *S. salsa* to light condition and different temperature regimes. Furthermore, *S. salsa* seeds used in germination studies are mainly from the humid regions and not mentioned the seed type (Song et al., 2008; Gao et al., 2018). Thus, germination characteristics of dimorphic seeds of *S. salsa* to different environmental factors and seedling growth are not well recorded, especially for this species grown in arid saline soils.

Light is an important environmental factor and the responses of seeds to it can control the time and place of seed germination (Pons, 2014). Light can promote or inhibit seed germination via light-mediated signaling network, and might has no significant effect (Yang et al., 2020). Non-dormant seeds are generally not sensitive to light during germination, whereas photoblastism is very common for seeds with non-deep physiological dormancy (Baskin and Baskin, 2014). Effects of light on seed germination depend on plant species and other environmental factors during germination (Qu et al., 2008; Nisar et al., 2019a; Yang and Chen, 2020). Even for seeds from the same species, the germination responses to light can be dramatically different. For example, light does not affect germination of non-dormant brown seeds of *Suaeda aralocaspica*, but promotes germination of cold-stratified black seeds, especially for black seeds after stratification in darkness (Wang et al., 2017).

Temperature is one of the most important environmental factors for change of seed dormancy and germination velocity (Fenner and Thompson, 2005). The temperature requirement for germination is determined by the plant species, source of the seeds, genetic differences with a given species, the age of the seeds, as well as by the seed position on a single plant (Gutterman, 2002; Bhatt et al., 2020). Generally, the temperature range for germination is wider for non-dormant seeds than that for dormant seeds (Baskin and Baskin, 2014). Furthermore, the interaction of light and temperature on germination is demonstrated in some species (Li et al., 2005; Wang et al., 2017). Therefore, studying the effects of light conditions, temperature range, and the interdependence between light and temperature on seed germination is essential to understand seed germination strategy of *S. salsa* in the field.

High soil salinity can inhibit seed germination via osmotic effect and toxic effect (Gul et al., 2013; Song et al., 2017). Although the toxic effects on the hydrated seeds of halophytes have been reported, the decline in germination caused by salinity is primarily osmotic effect (Rasheed et al., 2019). For example, the brown seeds of *S. aralocaspica* can germinate to 10% at 1400 mM NaCl. Furthermore, they are able to endure prolonged exposure to high concentration of NaCl (4000 mM) and germinate normally when salt stress is removed (Wang et al., 2008). Compared with adult plants, germinating seeds and young seedlings are more sensitive to salinity (Gul et al., 2013). However, the quantitative comparison of salt tolerance between germinating seeds and young seedlings is scarce. The ionic composition of the soil can also affect seed germination and seedling growth (Zhang et al., 2018). Salinity problems are mainly due to excess Na^+^ with Cl^–^ or SO_4_^2–^ as the counter ions in the arid northwestern China (Xi et al., 2006).

The aim of this study was to test the effects of light, temperature, and two types of salinity on germination of dimorphic seeds of *S. salsa*, and the effects of both types of salt on seedling growth. Specifically, we asked the following questions: (1) Do dimorphic seeds have the same light and temperature requirements during germination? (2) Which seed type has higher germination percentage and recovery germination under high salinity? (3) Do germinating seeds and thereafter seedlings have the same salt tolerance? The findings will provide essential information for understanding the role of different environmental factors in regulating germination of dimorphic seeds and seedling growth of halophyte *S. salsa* in arid saline desert.

## Materials and Methods

### Plant Materials

Freshly mature fruits of *S. salsa* were randomly collected from the population growing in a salt desert of Karamay, northern Xinjiang, China in November 2019. The companion species are *Salicornia europaea*, *Salsola subcrassa*, *Phragmites australis*, and so on. This site belongs to temperate continental desert climate. The annual average temperature is 8.4°C. The mean annual precipitation is 109 mm and mean annual potential evaporation is 3009 mm (Baiketuerhan et al., 2019).

The fruits were allowed to dry naturally in laboratory room for 2 weeks. Seeds were cleaned and separately sorted according to seed color. Each type of seeds was pooled and stored in plastic bag at room temperature (22 ± 2°C) until used in experiments.

### Seed Germination

#### Effects of Light and Temperature on Germination

For each treatment, four biological replicates of 25 seeds were placed in a 50 mm diameter Petri dish on two layers of No. 1 filter paper moistened with 2.5 ml of deionized water. After covered with lid, the petri dish was sealed with parafilm and then transferred to plant incubator (GXZ-380, Jiangnan Instrument Factory, Zhejiang, China). The incubation temperature regimes were 5: 15°C, 5: 20°C, 10: 25°C, 15: 30°C, and 20: 35°C. The light condition was set to 12 h light/darkness photoperiod (hereafter light treatment) or constant darkness. For light treatment, higher temperature of each temperature regime was coincided with 12 h light and lower temperature with 12 darkness. Two layers of aluminum foil were used to wrap the Petri dishes to provide constant darkness. Seeds were incubated for 20 days. Germination test standard for black seeds was ≥1 mm radicle and for brown seeds it was ≥2 mm. Seeds under the light treatment were checked every 24 h, and germinated seeds were counted and discarded. Seeds under constant darkness were checked only after 20 days of incubation.

#### Effects of Salinity on Germination and Recovery

Analytical reagent grade of NaCl and Na_2_SO_4_ (Fuchen Chemical Reagent Co., Ltd., Tianjin, China) were used to study the effects of salinity on germination and recovery of dimorphic seeds of *S. salsa*. For each treatment, five biological replicates of 25 seeds were incubated in Petri dishes on two layers of filter paper moistened with 2.5 ml solution (0, 50, 100, 200, 300, 400,600, 800, 1000, 1200 mM of NaCl and Na_2_SO_4)_. Then the Petri dishes were placed under 10: 25°C and light treatment for 20 days. Based on the first experiment, 10:25°C is suitable for germination. Furthermore, it represents the temperature of May and September in this area. The Petri dishes were checked daily.

Ungerminated seeds in each Petri dish were rinsed three times with deionized water and then incubated for 5 days in a new Petri dish that filled with 2.5 ml deionized water under the above mentioned temperature and light conditions. Recovery germination was checked every day.

Germination percentage (%) = (the number of seeds that germinated in each solution/total number of seeds tested in in each solution) × 100. Recovery percentage (%) = (the number of seeds germinated in recovery experiment in each solution/the number of ungerminated seeds in each solution) × 100. Final germination percentage (%) = (total number of seeds that germinated in each solution plus those that recovered to germinate in deionized water/the total number of seeds tested in each solution) × 100. The velocity of germination was estimated using a modified Timson’s index of germination velocity (Khan et al., 2001).

### Seedling Growth

During the germination process under different salinity, seven germinated seeds in each Petri dish were selected according to the germination time. Then, they were incubated in a new Petri dish with the same salt treatment under 10: 25°C and light treatment for 20 days. Radicle length and shoot length were measured for the two longest seedlings in each Petri dish by the microscope with Olumpus cellSens software. Thus, there were 10 replicates for each treatment. Radicle tolerance index (%) = (length of the radicle in different salt treatments/length of the radicle under deionized water treatment) × 100. Shoot tolerance index (%) = (length of the shoot in different salt treatments/length of the shoot under deionized water treatment) × 100.

### Data Analysis

All data were expressed as mean ± SE. The data did not meet the assumptions for three-way ANOVA. In light and temperature experiment, data for germination percentage were analyzed by linear regression using the linear regression method (all independent variables were entered into the equation in a single step). The multiple linear regression model included seed type (brown and black seeds), light condition (12 h light/darkness photoperiod and constant darkness), and temperature regimes (5: 15°C, 5: 20°C, 10: 25°C, 15: 30°C, and 20: 35°C). These thermoperiods represent the mean daily maximum and minimum monthly temperatures at the research field during the growing season: 5:15 (early April and October), 5:25 (late April), 10:25 (May and September), 15:30 (June and August), and 20:35°C (July). In salinity experiment, data for germination and seedling growth were also analyzed by linear regression. The multiple linear regression model included seed type (brown and black seeds), salt type (NaCl and Na_2_SO_4_), and salt concentration (50, 100, 200, 300, 400, 600, 800, 1000, and 1200 mM). One-way ANOVA and Tukey’s test were used to determine significant differences among different temperatures or salinity treatments for each seed type of *S. salsa*. Independent samples *t*-test was used to determine whether there was difference between brown and black seeds under the same conditions.

## Results

### Seed Germination

#### Effects of Light and Temperature on Germination

**FIGURE 1:**
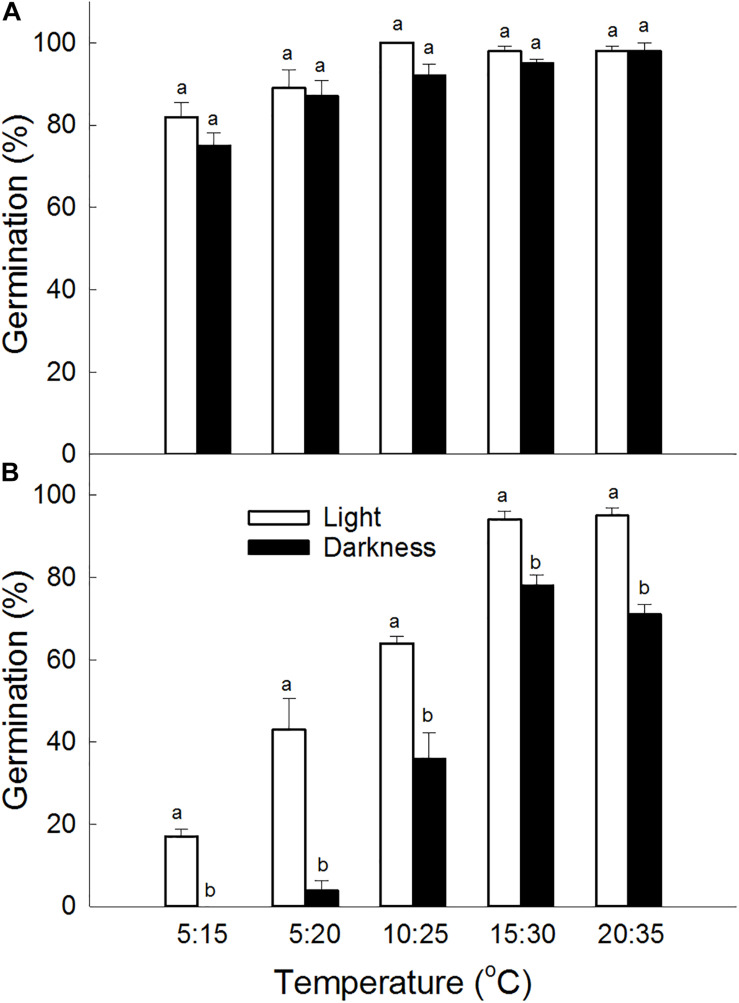
Effect of light condition and temperature regime on germination percentage of **(A)** brown and **(B)** black seeds of *S. salsa*. For each treatment there were four biological replicates. Different lower-case letters indicate significant differences in germination percentages between dimorphic seeds at the same temperature.

Germination percentage was significantly affected by seed type (*P* < 0.001), light condition (*P* < 0.001), and temperature (*P* < 0.001). Germination percentages of brown seeds of *S. salsa* were higher than that of black seeds under the same conditions (Figure 1). There was no significant difference in germination percentage between incubation in light and incubation in darkness for brown seeds. However, for black seeds, germination percentage in light was significantly higher than that in darkness under the same temperature. Although temperature significantly (*P* < 0.001) affected germination of brown seeds, germination percentages in different temperature combinations at 5: 20°C, 10: 25°C, 15: 30°C, and 20: 35°C did not show significantly differences. For black seeds, germination percentages increased as the temperature increased from 5: 15°C to 15: 30°C (Figure 1).

#### Effects of Salinity on Germination and Recovery

Germination percentage and germination index were significantly affected by seed type (*P* < 0.001), salt type (*P* < 0.001) and salt concentration (*P* < 0.001) (Table 1). Recovery percentage was significantly affected by seed type (*P* < 0.001) and salt concentration (*P* < 0.001). Final germination percentage was significantly affected by salt type (*P* = 0.002) and salt concentration (*P* < 0.001) (Table 1).

**TABLE 1:** Multiple linear regression analysis of germination percentage, germination index, recovery percentage, and final germination percentage of dimorphic seeds, and radicle and shoot length of seedlings grown from dimorphic seeds of *S. salsa*.

		B (SE)	β	*P*-value
Germination percentage	Seed type	−20.92 (1.855)	−0.295	<0.001
	Salt type	−12.44 (1.855)	−0.176	<0.001
	Salt concentration	−0.077 (0.002)	−0.865	<0.001
Germination index	Seed type	−37.198 (1.933)	−0.536	<0.001
	Salt type	−9.442 (1.933)	−0.136	<0.001
	Salt concentration	−0.064 (0.002)	−0.737	<0.001
Recovery percentage	Seed type	0.038 (0.003)	0.603	<0.001
	Salt type	1.958 (2.028)	0.039	0.336
	Salt concentration	27.961 (2.028)	0.559	<0.001
Final germination percentage	Seed type	1.280 (2.839)	0.025	0.653
	Salt type	−8.960 (2.839)	−0.176	0.002
	Salt concentration	−0.039 (0.004)	−0.601	<0.001
Shoot length	Seed type	−2.526 (0.173)	−0.343	<0.001
	Salt type	−1.174 (0.173)	−0.159	<0.001
	Salt concentration	−0.007 (0)	−0.799	<0.001
Radicle length	Seed type	−2.472 (0.282)	−0.255	<0.001
	Salt type	−0.324 (0.282)	−0.033	0.252
	Salt concentration	−0.009 (0)	−0.774	<0.001

**FIGURE 2:**
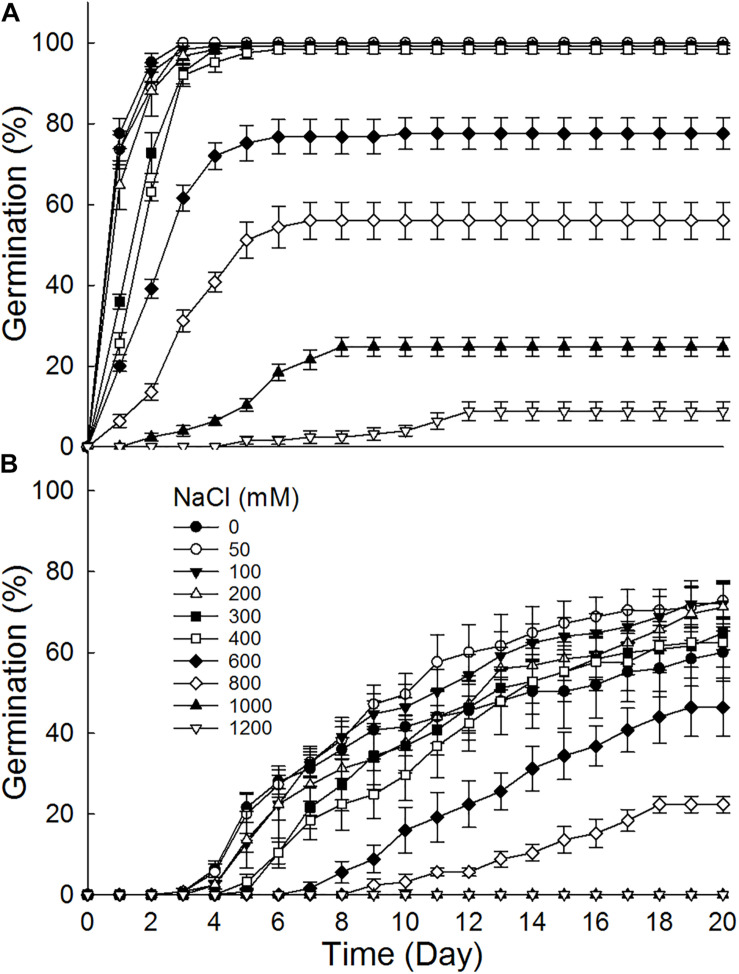
Effect of NaCl on germination of **(A)** brown and **(B)** black seeds of *S. salsa* incubated at 10:25°C in the 12 h daylight photoperiod for 20 days. For each treatment there were five biological replicates.

**FIGURE 3:**
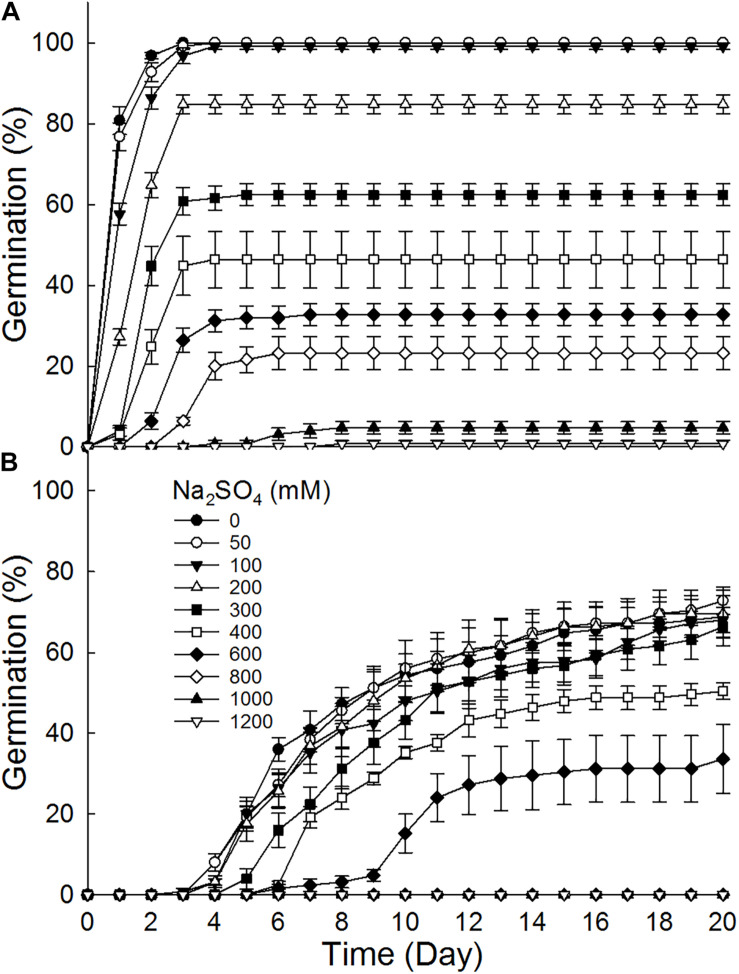
Effect of Na_2_SO_4_ on germination of **(A)** brown and **(B)** black seeds of *S. salsa* incubated at 10:25°C in the 12 h daylight photoperiod for 20 days. For each treatment there were five biological replicates.

Brown seeds germinated to 8.8–100% at 0–1200 mM NaCl, while black seeds germinated to 0–72% at 0–1000 mM (Figure 2). Furthermore, brown seeds germinated to higher percentages than black seeds at the same NaCl concentration. Germination percentage of brown seeds at 800 mM NaCl was 56%, whereas that of black seeds was only 22.4%. The increase of NaCl concentration gradually decreased germination percentages of dimorphic seeds. Dimorphic seeds in Na_2_SO_4_ solutions showed a different pattern in germination (Figure 3). Brown seeds had higher germination percentage than black seeds at low and high Na_2_SO_4_ concentrations. However, dimorphic seeds had similar germination percentage at middle concentration (300–600 mM) of Na_2_SO_4_. The increase of Na_2_SO_4_ concentration also decreased germination of both types of seeds. At the same salt concentration, brown seeds generally had higher germination percentage in NaCl solution than that in Na_2_SO_4_ solution. At the relatively high concentration (400–800 mM), black seeds had higher germination percentage in NaCl than that in Na_2_SO_4_ solution (Figures 2, 3).

Germination indexes for dimorphic seeds under different salinity had the similar trend as germination percentage (Table 2). But the difference of germination index between dimorphic seeds was more significant than that of germination percentage. Germination index of brown seeds was higher than that of black seeds at the same salt concentration. The highest gemination index for brown seeds in NaCl solutions was 98.6, whereas that for black seeds was 44.3. The highest gemination index for brown seeds in Na_2_SO_4_ solutions was 98.9, but that for black was 45.2.

**TABLE 2:** Germination index of dimorphic seeds of *S. salsa* under different NaCl and Na_2_SO_4_ concentration.

Concentration (mM)	NaCl		Na_2_SO_4_	
	Brown seed	Black seed	Brown seed	Black seed
0	98.6 ± 0.3*Aa*	36.3 ± 5.3*Ba*	98.9 ± 0.2*Aa*	44.7 ± 3.4*Ba*
50	98.1 ± 0.2*Aa*	44.3 ± 4.0*Ba*	98.4 ± 0.2*Aa*	45.2 ± 3.3*Ba*
100	97.6 ± 0.9*Aa*	41.8 ± 3.5*Ba*	96.4 ± 0.8*Aa*	40.6 ± 3.4*Bab*
200	96.8 ± 0.7*Aa*	38.0 ± 4.9*Ba*	80.9 ± 2.3*Ab*	43.9 ± 4.4*Ba*
300	94.4 ± 0.8*Aa*	34.2 ± 3.6*Ba*	58.5 ± 2.7*Ac*	36.8 ± 3.3*Bab*
400	92.5 ± 1.1*Aa*	32.3 ± 4.7*Bab*	43.1 ± 6.5*Ad*	28.8 ± 1.7*Ab*
600	71.4 ± 3.5*Ab*	19.0 ± 3.4*Bbc*	29.4 ± 2.5*Ae*	16.3 ± 4.2*Bc*
800	49.1 ± 3.7*Ac*	7.5 ± 1.1*Bcd*	20.3 ± 3.6*Ae*	0.0 ± 0.0*Bd*
1000	19.3 ± 1.7*Ad*	0.0 ± 0.0*Bd*	4.5 ± 1.2*Af*	0.0 ± 0.0*Bd*
1200	5.0 ± 1.4*Ae*	0.0 ± 0.0*Bd*	0.5 ± 0.5*Af*	0.0 ± 0.0*Ad*

*For each treatment there were five biological replicates. Different lower-case letters indicate significant differences (P < 0.05, Tukey’s test) in germination indexes among different concentration for the same seed type and salt type. Different upper-case letters indicate significant differences (P < 0.05, Tukey’s test) between dimorphic seeds at the same concentration of NaCl or Na_2_SO_4_.*

Germination recovery percentage of brown seeds was lower than that of black seeds after the pretreatment with the same salt concentration (Table 3). Recovery percentages of brown seeds were 0 after pretreatment with 0–600 mM NaCl or Na_2_SO_4_. After pretreatment with high NaCl concentration (800–1200 mM), recovery percentages of brown seeds were 3.3–9.6%, however after pretreatment with high Na_2_SO_4_ concentration, recovery percentages of brown seeds were 1.2–17.0%. As the pretreatment salinity increased, the recovery percentages of black seeds showed a clear increasing trend. As the concentration of pretreated NaCl increased from 0 to 1200 mM, the recovery percentage of black seeds increased from 0 to 79.2%. Similarly, as the pretreatment Na_2_SO_4_ increased from 0 to 1200 mM, the recovery percentage of black seeds increased from 4.5 to 80.8% (Table 3).

**TABLE 3:** Recovery percentage of dimorphic seeds of *S. salsa* in deionized water after NaCl or Na_2_SO_4_ pretreatment.

Concentration (mM)	NaCl		Na_2_SO_4_	
	Brown seed	Black seed	Brown seed	Black seed
0	0.0 ± 0.0*Ac*	2.0 ± 2.0*Af*	0.0 ± 0.0*Ac*	4.5 ± 2.9*Ac*
50	0.0 ± 0.0*Ac*	2.0 ± 2.0*Af*	0.0 ± 0.0*Ac*	2.9 ± 2.9*Ac*
100	0.0 ± 0.0*Ac*	0.0 ± 0.0*Af*	0.0 ± 0.0*Ac*	4.3 ± 2.7*Ac*
200	0.0 ± 0.0*Ac*	16.2 ± 5.9*Aef*	0.0 ± 0.0*Ac*	7.8 ± 4.8*Ac*
300	0.0 ± 0.0*Ac*	20.1 ± 8.7*Adef*	0.0 ± 0.0*Bc*	19.3 ± 5.5*Ac*
400	0.0 ± 0.0*Bc*	27.2 ± 7.7*Acde*	0.0 ± 0.0*Bc*	18.8 ± 4.2*Ac*
600	0.0 ± 0.0*Bc*	42.9 ± 6.8*Abc*	0.0 ± 0.0*Bc*	42.7 ± 6.9*Ab*
800	3.3 ± 3.3*Bbc*	41.1 ± 3.0*Abcd*	1.2 ± 1.2*Bc*	62.4 ± 3.7*Aa*
1000	7.3 ± 1.1*Bab*	54.4 ± 4.8*Ab*	6.8 ± 1.0*Bb*	76.0 ± 4.7*Aa*
1200	9.6 ± 1.6*Ba*	79.2 ± 3.2*Ba*	17.0 ± 2.8*Ba*	80.8 ± 6.4*Aa*

*For each treatment there were five biological replicates. Different lower-case letters indicate significant differences (P < 0.05, Tukey’s test) in recovery percentages among different concentration for the same seed type and salt type. Different upper-case letters indicate significant differences (P < 0.05, Tukey’s test) between dimorphic seeds at the same concentration of NaCl or Na_2_SO_4_.*

The final germination percentages of brown seeds were significantly higher than that of black seeds at 0–400 mM NaCl (Table 4). However, the final germination percentages of brown seeds were significantly lower than that of black seeds at 1000–1200 mM NaCl. As NaCl concentration increased from 0 to 1200 mM, final germination percentages of brown seeds decreased from 100 to 17.6%. But the final germination percentages of black seeds maintained a high level. For Na_2_SO_4_ treatments, brown seeds had higher final germination percentages than black seeds at low salinity (0–200 mM). But at high salinity (300–1200 mM), brown seeds had lower final germination percentages than black seeds. Final germination percentages of brown seeds also showed a clear decreasing trend under Na_2_SO_4_ treatments. When the concentration of salinity was 200–1000 mM, the brown seeds with NaCl treatment had higher final germination percentages than brown seeds with the same Na_2_SO_4_ concentration.

**TABLE 4:** Final germination percentage of dimorphic seeds of *S. salsa* after treated with different concentration of NaCl or Na_2_SO_4_.

Concentration (mM)	NaCl		Na_2_SO_4_	
	Brown seed	Black seed	Brown seed	Black seed
0	100.0 ± 0.0*Aa*	60.8 ± 7.2*Bab*	100.0 ± 0.0*Aa*	70.4 ± 4.8*Bab*
50	100.0 ± 0.0*Aa*	73.6 ± 4.1*Bab*	100.0 ± 0.0*Aa*	73.6 ± 3.5*Bab*
100	99.2 ± 0.8*Aa*	72.0 ± 5.8*Bab*	99.2 ± 0.8*Aa*	69.6 ± 2.4*Bab*
200	99.2 ± 0.8*Aa*	76.0 ± 5.5*Bab*	84.8 ± 2.3*Ab*	72.8 ± 4.5*Bab*
300	99.2 ± 0.8*Aa*	72.0 ± 4.6*Bab*	62.4 ± 2.7*Bc*	73.6 ± 3.5*Aab*
400	98.4 ± 1.0*Aa*	72.8 ± 4.1*Bab*	46.4 ± 7.0*Ac*	60.0 ± 1.3*Ab*
600	77.6 ± 3.9*Ab*	68.0 ± 7.2*Aab*	32.8 ± 2.7*Bd*	63.2 ± 4.5*Aab*
800	57.6 ± 4.5*Ac*	54.4 ± 2.0*Ab*	24.8 ± 4.8*Bde*	62.4 ± 3.7*Aab*
1000	30.4 ± 1.6*Bd*	54.4 ± 4.8*Ab*	12.8 ± 1.5*Be*	76.0 ± 4.7*Aab*
1200	17.6 ± 2.0*Be*	79.2 ± 3.2*Aa*	17.6 ± 3.2*Be*	80.8 ± 6.4*Aa*

*For each treatment there were five biological replicates. Different lower-case letters indicate significant differences (P < 0.05, Tukey’s test) in final germination percentages among different concentration for the same seed type and salt type. Different upper-case letters indicate significant differences (P < 0.05, Tukey’s test) between dimorphic seeds at the same concentration of NaCl or Na_2_SO_4_.*

### Seedling Growth

Shoot lengths were significantly affected by seed type (*P* < 0.001), salt type (*P* < 0.001), and salt concentration (*P* < 0.001). However, radicle lengths were significantly affected by seed type (*P* < 0.001) and salt concentration (*P* < 0.001) (Table 1).

**FIGURE 4:**
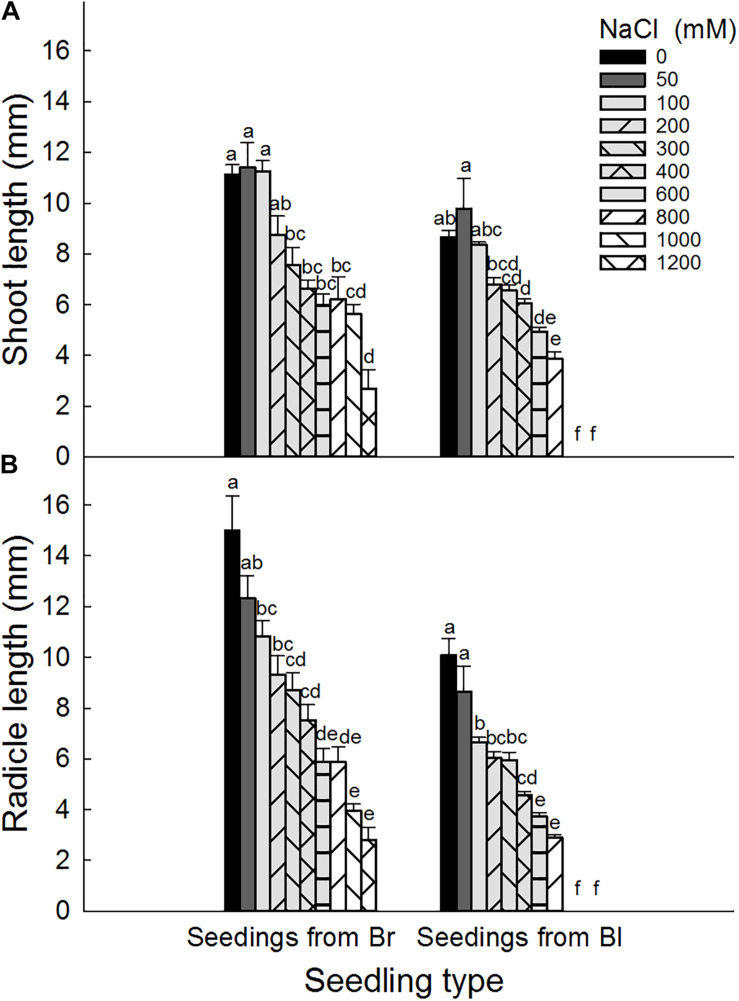
Effect of NaCl on **(A)** shoot and **(B)** radicle length of seedlings grown from dimorphic seeds of *S. salsa*. Br, brown seeds; Bl, black seeds. For each treatment there were 10 biological replicates. Different lower-case letters indicate significant differences in shoot or radicle lengths of seedlings grown from each seed type.

**FIGURE 5:**
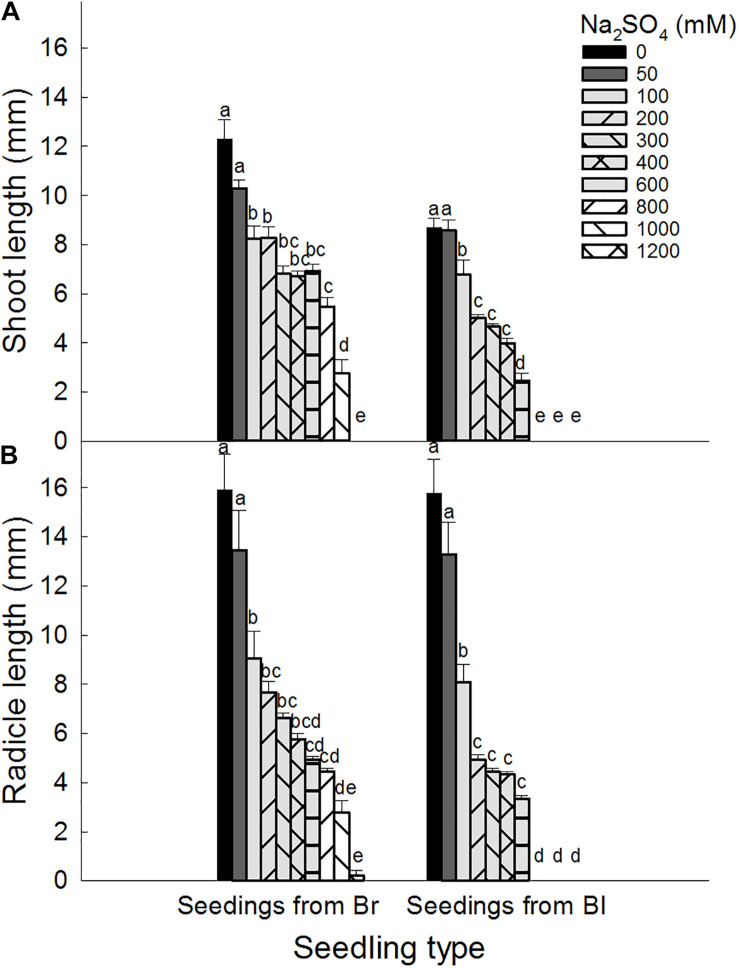
Effect of Na_2_SO_4_ on **(A)** shoot and **(B)** radicle length of seedlings grown from dimorphic seeds of *S. salsa*. Br, brown seeds; Bl, black seeds. For each treatment there were 10 biological replicates. Different lower-case letters indicate significant differences in shoot or radicle lengths of seedlings grown from each seed type.

**FIGURE 6:**
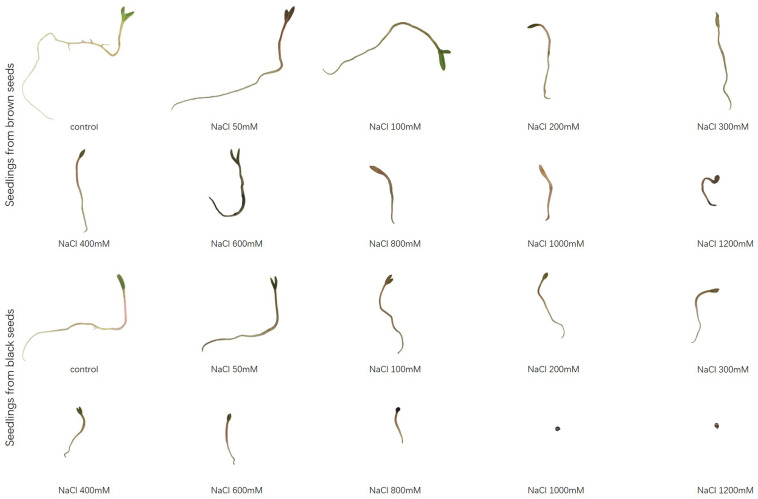
Radicle elongation of seedlings grown from dimorphic seeds of *S. salsa* after 20 days of incubation under different concentrations of NaCl.

**FIGURE 7:**
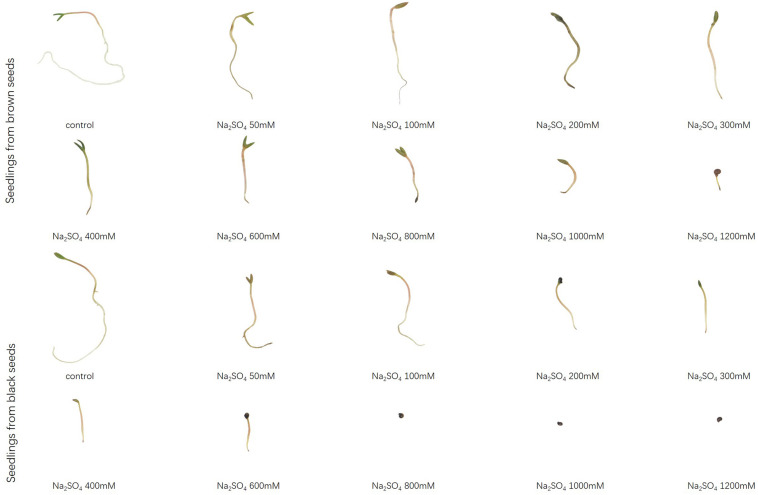
Radicle elongation of seedlings grown from dimorphic seeds of *S. salsa* after 20 days of incubation under different concentrations of Na_2_SO_4_.

Brown seeds were generally had higher length of shoot and radicle than black seeds at the same NaCl or Na_2_SO_4_ concentration (Figures 4, 5). At the same concentration, seedlings grown from the same type of seeds generally had higher length and better growth in NaCl solution than that in Na_2_SO_4_ solution. Radicle length of brown seeds was 2.8 ± 0.5 mm at 1200 mM NaCl solution, whereas only 0.2 ± 0.2 mm at the same concentration of Na_2_SO_4_. Shoot and radicle length of seedlings grown from dimorphic seeds generally deceased with the increase of salinity. Radicle growth of black seeds was almost completely inhibited by 1000 mM NaCl, and by 800 mM Na_2_SO_4_ (Figures 6, 7).

Shoot and radicle tolerance index showed similar trends in different salt solutions as the changes of radicle and shoot length (Tables 5, 6). Both indexes were generally higher in NaCl solution than that in the same Na_2_SO_4_ concentration. Radicle tolerance index was more sensitive to salt toxicity than shoot tolerance index. For example, the radicle tolerance index was 57.8 for seedlings grown from black seeds in 200 mM Na_2_SO_4_ solutions. However, the shoot tolerance index was 31.3 (Tables 5, 6).

**TABLE 5:** Shoot tolerance index of *S. salsa* seedlings grown from dimorphic seeds treated with different concentration of NaCl or Na_2_SO_4_.

Concentration (mM)	NaCl		Na_2_SO_4_	
	Seedlings from brown seeds	Seedlings from black seeds	Seedlings from brown seeds	Seedlings from black seeds
0	100.0 ± 3.4*Aa*	100.0 ± 2.5*Aab*	100.0 ± 6.5*Aa*	100.0 ± 4.0*Aa*
50	102.3 ± 9.0*Aa*	122.6 ± 15.0*Aa*	83.8 ± 2.9*Ba*	98.5 ± 4.8*Aa*
100	101.1 ± 3.8*Aa*	104.5 ± 1.5*Aab*	67.0 ± 4.4*Ab*	77.8 ± 6.8*Ab*
200	78.4 ± 6.8*Aab*	84.9 ± 3.3*Abc*	67.4 ± 3.7*Ab*	57.8 ± 1.5*Bc*
300	67.8 ± 6.4*Abc*	82.0 ± 2.9*Abc*	55.5 ± 2.6*Abc*	53.7 ± 1.1*Ac*
400	59.4 ± 3.3*Bbc*	75.6 ± 2.4*Ac*	54.8 ± 1.6*Abc*	45.9 ± 2.1*Bc*
600	53.8 ± 3.8*Abc*	61.7 ± 2.1*Acd*	56.3 ± 2.2*Abc*	28.2 ± 3.6*Bd*
800	55.9 ± 7.8*Abc*	48.4 ± 3.5*Ad*	44.7 ± 2.9*Ac*	0.0 ± 0.0*Be*
1000	50.5 ± 3.4*Acd*	0.0 ± 0.0*Be*	22.6 ± 4.5*Ad*	0.0 ± 0.0*Be*
1200	24.2 ± 6.8*Ad*	0.0 ± 0.0*Be*	0.0 ± 0.0*Aef*	0.0 ± 0.0*Ae*

*For each treatment there were 10 biological replicates. Different lower-case letters indicate significant differences (P < 0.05, Tukey’s test) in shoot tolerance indexes among different concentration for the same seed type and salt type. Different upper-case letters indicate significant differences (P < 0.05, Tukey’s test) between dimorphic seeds at the same concentration of NaCl or Na_2_SO_4_.*

**TABLE 6:** Radicle tolerance index of *S. salsa* seedlings grown from dimorphic seeds treated with different concentration of NaCl or Na_2_SO_4_.

Concentration (mM)	NaCl		Na_2_SO_4_	
	Seedlings from brown seeds	Seedlings from black seeds	Seedlings from brown seeds	Seedlings from black seeds
0	100.0 ± 9.0*Aa*	100.0 ± 5.4*Aa*	100.0 ± 9.1*Aa*	100.0 ± 8.7*Aa*
50	82.2 ± 6.0*Aab*	79.2 ± 9.0*Ab*	84.5 ± 10.2*Aa*	84.2 ± 8.3*Aa*
100	72.2 ± 4.2*Abc*	60.9 ± 1.8*Bc*	57.0 ± 6.9*Ab*	51.2 ± 4.7*Ab*
200	62.0 ± 5.0*Abc*	55.2 ± 2.4*Acd*	48.3 ± 2.6*Abc*	31.3 ± 1.3*Bc*
300	58.1 ± 4.5*Acd*	54.4 ± 2.9*Acd*	41.8 ± 1.2*Abc*	28.2 ± 0.8*Bc*
400	50.2 ± 4.0*Acd*	41.9 ± 1.2*Ade*	36.2 ± 1.6*Abcd*	27.4 ± 0.7*Bc*
600	39.1 ± 3.7*Ade*	34.1 ± 1.4*Ae*	31.0 ± 0.9*Acd*	21.2 ± 0.8*Bc*
800	39.1 ± 4.0*Ade*	26.5 ± 1.0*Be*	27.9 ± 0.8*Acd*	0.0 ± 0.0*Bd*
1000	26.4 ± 1.7*Ae*	0.0 ± 0.0*Bf*	17.5 ± 3.1*Ade*	0.0 ± 0.0*Bd*
1200	18.7 ± 3.3*Ae*	0.0 ± 0.0*Bf*	1.4 ± 1.4*Ae*	0.0 ± 0.0*Ad*

*For each treatment there were 10 biological replicates. Different lower-case letters indicate significant differences (P < 0.05, Tukey’s test) in radicle tolerance indexes among different concentration for the same seed type and salt type. Different upper-case letters indicate significant differences (P < 0.05, Tukey’s test) between dimorphic seeds at the same concentration of NaCl or Na_2_SO_4_.*

## Discussion

Although the effects of abiotic factors on seed germination of *S. salsa* have been previously reported (Song et al., 2008; Li et al., 2016; Zhang et al., 2020), our study is the first to test the difference of salt tolerance between seeds and seedlings, and the interactions of light and temperature on germination of dimorphic seeds of *S. salsa*. Our study shows that dimorphic seeds have differential germination responses to light and temperature conditions. Brown seeds are more tolerant to salinity than black seeds during germination and seedling growth. Na_2_SO_4_ is more harmful to the germinating seeds than NaCl. The results indicate that though brown seeds can germinate in high salt concentration, seedlings grown from them are damaged. Furthermore, recovery germination is an important strategy for black seeds after salt stress release.

Light did not affect germination percentages of brown seeds of *S. salsa* under different temperature regimes, however significantly improve germination of black seeds. The results suggest that black seeds of *S. salsa* require light to reach high germination percentages, and more sensitive to light than brown seeds. The differential responses of dimorphic seeds to light are also reported in *S. aralocaspica* after cold stratification (Wang et al., 2017). Germination of brown seeds of *S. aralocaspica* was not affected by light conditions, however germination of cold-stratified black seeds was improved by light. The light requirement for germination of dormant seeds might be fulfilled by a period of cold stratification or high incubation temperature (Wang et al., 2008; Baskin and Baskin, 2014). Light requirement for germination is also reported in other heteromorphic seeds. Both central and peripheral achenes of *Bidens pilosa* had higher germination percentages in light condition than in darkness (Zhang et al., 2019).

Temperature is an important environmental factor and differentially regulates germination of dimorphic seeds of *S. salsa* (Li et al., 2008). Brown seeds could germinate to high percentages (75%) in all of tested temperature regimes. This indicates that temperature is not a limiting factor for germination of brown seeds from spring to autumn and water conditions in the field might be a crucial factor. However, germination percentages of black seeds in light gradually increased from 17% at 5:15°C to 95% at 20:35°C, and in darkness increased from 0% at 5:15°C to 78% at 15:30°C. Our results show that germination of black seeds reach the highest at high temperature in light. The result reveals that black seeds of *S. salsa* have conditional dormancy and high temperature can decrease the limitation of dormancy during germination.

Salinity have different effects on germination and recovery of dimorphic seeds of *S. salsa*. Although germination percentages of dimorphic seeds decreased with the increase of salinity, brown seeds had higher salt tolerance than black seeds. At 600 mM NaCl, brown seeds germinated to 77.6%, but black seeds germinated to 46.4%. This might be due to the difference in antioxidant ability between dimorphic seeds (Nisar et al., 2019b). Ungerminated seeds under high salinity stress regerminated when transferred to deionized water, especially for black seeds. The germination recovery percentage of black seeds gradually increased with the increase of pretreated salinity. This reveals that high salt pretreatment just temporally inhibits the germination of black seeds. This phenomenon has also been reported in many halophytes (Wang et al., 2008; Cao et al., 2012). For example, *Limonium tabernense* seeds have relatively low germination under 400 mM NaCl, and ungerminated seeds have high germination recovery percentage (>80%) when transferred to distilled water (Fernández et al., 2016). Furthermore, Na_2_SO_4_ had a stronger inhibitory effect on seed germination of *S. salsa* than NaCl at the same concentration. A previous study also shows that germination inhibition of *S. salsa* is in the following order: Na_2_SO_4_ > Na_2_CO_3_ > NaCl (Duan et al., 2007). However, seedling growth rate of *Haloxylon ammodendron* was higher in treatment with Na_2_SO_4_ than in iso-osmotic treatment with NaCl. All radicles of *Kalidium caspicum* die before their length exceeded 5 mm in −0.8 MPa NaCl solution, but 95% of the emerging radicles survives beyond 5 mm in the iso-osmotic PEG treatment. These results indicate that we need to carry out iso-osmotic experiment on seed germination and seedling growth of *S. salsa* in future research.

Shoot and radicle length of brown seeds of *S. salsa* are generally longer than that of black seeds under the same salinity. Maybe this is because brown seeds are heavier, and germinate earlier than black seeds (Song et al., 2008). Furthermore, brown seeds had higher radicle and shoot tolerance indexes than black seeds at high salinity. However, seedlings grown from brown seeds are damaged in high salt solutions. This strategy is a high-risk strategy (Venable, 1985; Imbert, 2002; Liu et al., 2018). In contrast, seedlings grown from black seeds had low radicle and shoot tolerance indexes. This can protect seedlings from black seeds against salt stress. Seedlings grown from black seeds of *S. salsa* emerge after the soil salt concentration decreases in the field. This strategy is a low-risk strategy. Dimorphic seeds and seedlings of *S. salsa* take advantage of this bet-hedging strategy in harsh saline habitats.

**FIGURE 8:**
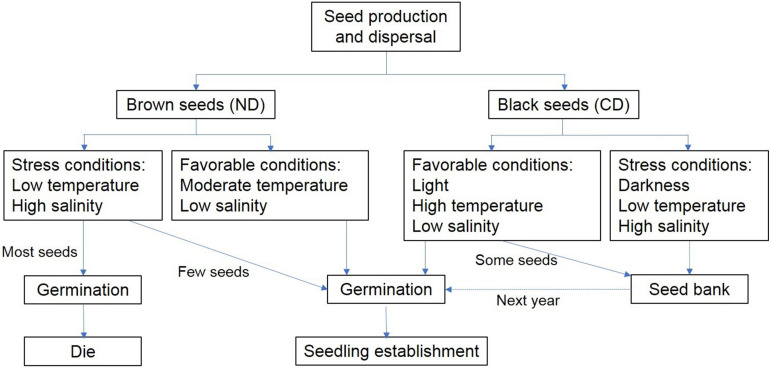
Conceptual model of dynamics of germination and seedling growth of dimorphic seeds of *S. salsa*.

Based on the pattern of germination of dimorphic seeds and seedling growth of *S. salsa*, we propose a conceptual model for their dynamics (Figure 8). Brown seeds are non-dormant and can germinate under high-stress conditions. However, high soil salinity leads to dramatic damage of seedlings grown from brown seeds. Early germination may also give plants a selective advantage (Gutterman, 2002). Black seeds perform differently from brown seeds. They have non-deep physiological dormancy when mature in fall, and become non-dormant after exposure to the cold temperature during early winter and early spring of next year. Then the state of black seeds are changed between non-dormant and conditionally dormant in the soil seedbank. In spring, low temperature and high salinity lead to low germination of black seeds, especially when exposure to the light. High precipitation in summer decreases soil salinity. High temperature and low salinity might lead to high germination of black seeds when in the shallow soil layer. Thus, seedlings grown from black seeds could avoid the salt damage. At the end of growing season, ungerminated seeds might enter the persistent soil seed bank. This bet-hedging strategy protects *S. salsa* from unpredictable disaster and maintain the population growth.

## Conclusion

In summary, the results indicate that dimorphic seeds of *S. salsa* have differential responses to light, temperature, and salinity. Light, temperature, and salinity are important environmental factors that regulating germination of black seeds. Brown seeds have high germination percentage and velocity in a wide range of environmental conditions. Seedlings of *S. salsa* are more sensitive than germinating seeds to the same salt concentration. Indexes of radicle can be used as an effective indicator to evaluate salt tolerance of halophytes. By using the bet-hedging strategy of dimorphic seeds, halophyte *S. salsa* can successfully adapt to the harsh saline deserts.

## Data Availability Statement

The raw data supporting the conclusions of this article will be made available by the authors, without undue reservation.

## Author Contributions

LW and JM contributed to the conception and design of the study. HZ, MH, and LJ collected the data. HZ, HM, LJ, ZZ, JM, and LW wrote the manuscript. HZ, MH, HM, LJ, ZZ, JM, and LW reviewed the manuscript. All authors have read and approved the final manuscript.

## Conflict of Interest

The authors declare that the research was conducted in the absence of any commercial or financial relationships that could be construed as a potential conflict of interest.
